# Recent advances in cancer surgery in older patients

**DOI:** 10.12688/f1000research.10683.1

**Published:** 2017-07-27

**Authors:** Siri Rostoft, Riccardo A. Audisio

**Affiliations:** 1 Institute of Clinical Medicine, University of Oslo, Oslo, Norway; 2 Department of Geriatric Medicine, Oslo University Hospital, Oslo, Norway; 3 St Helens Teaching Hospital Trust, University of Liverpool, Liverpool, UK

**Keywords:** cancer, surgery, older patients

## Abstract

Age is the most important risk factor for the occurrence of cancer, and a declining mortality from heart disease and other non-cancer causes leaves an older population that is at high risk of developing cancer. Choosing the optimal treatment for older cancer patients may be a challenge. Firstly, older age and associated factors such as comorbidities, functional limitations, and cognitive impairment are risk factors for adverse effects of cancer treatment. Secondly, older patients are often excluded from clinical trials, and current clinical guidelines rarely address how to manage cancer in patients who have comorbidities or functional limitations. The importance of incorporating frailty assessment into the preoperative evaluation of older surgical patients has received increasing attention over the last 10 years. Furthermore, studies that include endpoints such as functional status, cognitive status, and quality of life beyond the standard endpoints, i.e. postoperative morbidity and mortality, are starting to emerge. This review looks at recent evidence regarding geriatric assessment and frailty in older surgical cancer patients and provides a summary of newer studies in colorectal, liver, pancreatic, and gynecological cancer and renal and central nervous system tumors.

## Introduction

The majority of cancer patients are older, and, since surgery is one of the key treatment modalities in cancer, medical professionals need to keep up to date with recent advances in cancer surgery for older patients. Over the last 10 years, the importance of incorporating frailty assessment in the preoperative work-up for older surgical cancer patients has become evident since it has been shown that it successfully predicts the length of hospital stay, complications, survival, and costs ^1, 2^. Surgical techniques have become less invasive, anesthesia has become safer, and life expectancy is rising steadily; for this reason, octogenarians, nonagenarians, and even centenarians are being considered for major cancer surgery. There is thus a strong need for selecting older patients for the right treatment. Older patients are heterogeneous, ranging from fit patients with a long life expectancy to frail patients with a very short life expectancy, and chronological age alone is a poor predictor of life expectancy and treatment outcomes ^3– 5^. Lack of information to risk-stratify older patients for treatment may lead to under-treating fit older patients and over-treating frail older patients. In this review, we look at publications from the last five years on geriatric assessment (GA) and frailty in older surgical cancer patients. We will also present selected studies in colorectal, liver, pancreatic, and gynecological cancers as well as renal and central nervous system (CNS) tumors, with a particular focus on publications that include GA and frailty data.

## Methods

A literature search using the Medline database was performed. The following inclusion criteria were used: humans, review, adult, English language, and publication during the last five years. The search terms used were “((“Geriatric Assessment”[Mesh]) OR (geriatric assessment*[tiab])) AND ((“Neoplasms”[Mesh]) OR (neoplasm*[tiab] OR cancer*[tiab] OR tumor[tiab] OR tumors[tiab] OR oncolog*[tiab] OR malignan*[tiab])). Abstracts were reviewed for relevance to the review question. Other significant studies cited in the reference lists of the selected papers were also evaluated. We focused on papers regarding GA and frailty in cancer surgery as well as selected specific cancer types: colorectal, liver, pancreatic, and gynecological cancer as well as renal and CNS tumors. The literature search identified 170 citations, of which 42 were reviewed in detail, and 28 were included in this review.

## Geriatric assessment and frailty in cancer surgery

In recent years, four systematic reviews on frailty and GA in older surgical patients with cancer have been undertaken ^1, 6– 8^. Frailty is a term used as a marker of vulnerability, identifying individuals with a reduced capacity to respond to external stressors. Frailty may be viewed as a summary of a person’s health status and gives a more precise description of an individual’s vulnerability than does chronological age. In cancer surgery, there is no standardized way of identifying frailty in an individual patient. The two most commonly used methods are the physical frailty phenotype and the accumulation of deficits theory ^9^. Fried and colleagues constructed the physical frailty phenotype model, where the patient is assessed in five dimensions: weight loss, physical activity, exhaustion, grip strength, and walking speed ^10^. A person who scores below the 20th percentile of the normal population in at least three of these five dimensions is considered frail, while a person who scores poorly in two dimensions is categorized as pre-frail. The phenotypic frailty model is linked to sarcopenia (loss of muscle mass and function) and functional status but does not consider comorbidity and cognitive function. Rockwood and colleagues have suggested assessing frailty based on counting the number of deficits across a variety of health indicators such as functional status, cognitive function, comorbidities, emotional and nutritional status, and social support ^11^. The more that is wrong with the person, the frailer the person is. This model considers comorbidities and disability to be deficits associated with aging, and such deficits eventually lead to a physiological decline. In geriatric medicine, a GA is a systematic evaluation of an individual’s functional status, comorbidities, polypharmacy, cognitive status, nutritional status, emotional status, and social support (see Table 1). Based on the GA, the treating physician may quantify frailty, identify treatable conditions that can be optimized before surgery (such as malnutrition and depression), predict postoperative complications, evaluate decision-making capacity, and plan the treatment trajectory. Feng and colleagues reviewed prospective cohort studies published between 2000 and 2013 that looked at GA and postoperative outcomes in patients older than 60 years ^7^. The authors concluded from the review that components of the GA are consistently associated with adverse postoperative outcomes. Outcomes included postsurgical morbidity and mortality, discharge to an institution, and all-cause mortality. Six studies were identified: two thoracic oncology cohorts, three colorectal cancer (CRC) cohorts, and one cohort of solid tumors. The studies included various components of a GA, and the most frequently incorporated components were functional status measured by activities of daily living (ADLs), comorbidities, nutrition, and mood. The least-included components were mobility, frailty as a composite measure, polypharmacy, and social support. Postoperative complications were predicted by higher American Society of Anesthesiology (ASA) scores, cognitive impairment, functional impairment, depression, and frailty—regardless of how frailty was identified. None of the studies found that age was a predictor of complications. Handforth and colleagues studied the prevalence and outcomes of frailty in older cancer patients in a systematic review ^1^. The review included 20 studies; 3 of the studies were in surgical patients. The authors concluded that the prevalence of frailty and pre-frailty is high in older cancer patients; median estimates were42% and 43%, respectively. However, the definition of frailty varied considerably between studies. In any case, older cancer patients with frailty were at higher risk of all-cause mortality, postoperative mortality, and postoperative complications. Current data thus indicate three important issues: (1) a large number of older surgical cancer patients are frail or pre-frail, (2) frailty can be identified based on a GA, and (3) frailty is a predictor of negative outcomes, including 30-day postoperative morbidity and mortality as well as long-term mortality. Therefore, it is clear that frailty needs to be assessed routinely in older patients. In clinical practice, an assessment of frailty based on a GA may identify cognitive impairment relevant for decision-making, comorbidities, polypharmacy, and nutritional issues that can be optimized as well as functional status that is an important predictor of life expectancy, complications, and rehabilitation potential. In research, it is mandatory to include frail patients and describe patient frailty by including data regarding comorbidities and functional and cognitive status so that results from studies can be generalized to the real-world setting. If clinical trials keep failing to include patients with comorbidities and frailty, we will not be able to improve care of the real-world population of older surgical cancer patients ^12^. Unfortunately, as will be clear from the remainder of this review, only a few studies in older surgical cancer patients include frailty and GA data.

**Table 1.** Examples of domains and tests included in a geriatric assessment.

Domain	Tools (examples)
Functional status – Activities of daily living (ADL)	ADL (Katz index) IADLs (Lawton Scale)
Functional status – objective performance tests	5 m gait speed Timed Up and Go Short Physical Performance Battery
Comorbidity	Charlson’s comorbidity index Cumulative Illness Rating Scale
Polypharmacy	Number of drugs
Cognitive function	Mini-Cog™ Mini Mental State Evaluation
Nutritional status	Mini Nutritional Assessment
Emotional status	Geriatric Depression Scale
Social support	

IADL – instrumental activities of daily living

## Colorectal cancer

Several studies have reported on the use of GA and frailty to predict outcomes in older patients operated upon for CRC ^3, 5, 8^. A recent study from the Netherlands showed that the utilization of a laparoscopic approach in patients with CRC increased significantly between 2008 and 2011, resulting in reduced mortality rates, particularly for the older population. The reduction in mortality rates was shown for perioperative mortality, 3-month mortality, and 1-year mortality ^13^. Even though patients who were treated laparoscopically were younger and had lower disease stage and less comorbidity, the authors argued that the change in mortality was not the result of confounding by indication. Another review looked at long-term changes in physical function in older patients after surgery ^14^. It was found that both physical functioning and role functioning were significantly affected by CRC surgery. Initial losses were partially recovered during follow-up, but there seemed to be a permanent loss in physical capacity, especially in older patients, most commonly defined as 70 years and older. In 2014, the International Society of Geriatric Oncology (SIOG) published updated guidelines on the treatment of CRC in older patients ^15^. In these guidelines, the need for a pre-treatment GA was emphasized. Other recommendations regarding surgery included considering a prehabilitation program, avoiding emergency surgery, and carefully considering the consequences of the construction and siting of the stoma. Ugolini and colleagues reviewed the literature regarding CRC in older patients and pointed out that evidence-based guidelines are lacking, since older patients with comorbidities and frailty are often excluded from clinical trials ^16^. Data show that particularly preoperative frailty, comorbidity, and malnutrition are associated with negative outcomes. The review also emphasizes the good results that have been obtained with laparoscopic surgery in older patients with CRC. These results extend beyond improved postoperative morbidity, as functional recovery is also improved with laparoscopic treatment. The review by Ugolini recommends the fast-track programs in older patients operated upon for CRC, as this reduces morbidity and improves functional recovery. Studies show that older patients derive the same benefit from fast-track programs as do younger patients, even if the older cohort has more comorbidities and higher ASA scores.

In a prospective study from Norway, the changes in quality of life (QoL) after CRC surgery in frail patients compared to non-frail patients were evaluated ^17^. The study found a statistically and clinically significant improvement in QoL scores three months after surgery, both for the total cohort and for the subgroups of non-frail and frail patients. One-way repeated-measures analyses of variance showed a significant effect of time for emotional function ( *P*<0.001) and QoL ( *P*<0.001). For physical function, however, there was no overall significant effect of time ( *P*=0.08). The patients categorized as frail had lower scores overall (for physical function, the overall difference was 20.4), but the trajectory of scores was comparable to the non-frail group. At long-term follow-up (1.5–2.5 years), emotional functioning and QoL scores had decreased across groups but still remained above baseline values. Based on these results, being frail does not seem to be a contraindication to elective surgery, even if frailty predicts postoperative complications.

## Liver tumors and pancreatic cancer

Owing to advances in perioperative care and surgical techniques, an increasing number of older patients are offered resection of primary and secondary liver tumors. In a study from 2016, Sciergens and colleagues showed that patient age has limited impact on survival in the first 39 months after surgery ^18^. In their study, where comorbidities were assessed by using Charlson’s comorbidity index, comorbidity did not decrease survival within the first 5 years. Unfortunately, the study did not include data on patient frailty, and patients who underwent surgery were probably more fit than the general population. In comparison, a population-based study from Canada showed that older patient age was associated with not receiving liver surgery for CRC liver metastases: patients older than 75 underwent one resection per 101 incident cases, while patients younger than 65 underwent one resection per 26 incident cases ^19^.

Pancreatic cancer is an aggressive form of cancer, and the prognosis is poor. Changes in perioperative management have allowed for resections in older patients if they are fit. Studies of octogenarians undergoing pancreatic surgery are usually small but show that selected patients may undergo surgery with postoperative results comparable to younger patients ^20^. Riall and colleagues investigated time trends in surgical resection rates and operative mortality in older adults with locoregional pancreatic cancer in order to determine the effect of age on surgical resection rates and survival ^21^. From 1992 to 2005, surgical resection rates in patients aged 66 and older increased from 20 to 29%, respectively. Postoperative mortality rates decreased from 9 to 5%. Patients were less likely to be resected with older age, but, even for patients over 85 years, resection was associated with lower hazard of death compared to unresected participants younger than 70 years. The authors concluded that the benefit of resection does not diminish with older age in *selected* patients. In a systematic review and meta-analysis from 2014 looking at pancreatic resection in patients aged 80 and older, Casadei and colleagues concluded that patients over the age of 80 have an increased incidence of postoperative mortality, morbidity, and cardiac complications, and they advised that only selected patients should undergo surgery ^22^. However, comorbidities seem to be responsible for the increased risk associated with surgery in older patients. Unfortunately, patient frailty was not reported in any of the nine studies included in the review, and all studies were retrospective. In conclusion, older age seems to be a predictor of negative outcomes for major surgery such as pancreatic resections, but the data are limited. For colorectal cancer resections, the impact of age per se seems to be less pronounced.

## Gynecological cancer

Older women with cervical cancer experience poorer survival than do younger women, and a review by Elit and colleagues tries to answer why this is the case ^23^. The authors find that a number of confounders influence whether age impacts survival, such as comorbidities, stage, histology, grade, type of treatment, less radical surgery, lack of adjuvant radiation therapy, and lower rates of chemotherapy. Studies show that when older women are treated in a similar fashion to younger women, survival is comparable. However, this is partly because older women in such studies are selected and do not represent the normal population. Again, studies that include GA variables are needed.

In ovarian cancer, primary cytoreductive surgery followed by platinum-based chemotherapy is the cornerstone of treatment in patients with advanced disease. Women over the age of 80 are less likely to receive surgery and less likely to have an optimal cytoreduction ^24^. There is substantial toxicity associated with aggressive primary cytoreduction in older patients, particularly those over the age of 75. In a recent systematic review, both increased age and disease stage were strong predictors of 30-day mortality ^25^. The authors found that the estimated 30-day mortality for primary cytoreduction was 4.6%. In patients older than 75 with FIGO stage IV disease, the 30-day predicted mortality reached almost 40%. In a retrospective study of 85 patients over the age of 80 undergoing cytoreductive surgery, 13% died prior to discharge and 20% died within 60 days of surgery ^26^. In a pragmatic paper on the treatment of ovarian cancer in older women, Dr Lichtman proposes how to approach decision-making ^27^. The surgeon should consider two factors: firstly, if the disease is anatomically amenable to optimal resection, and, secondly, whether the patient is able to tolerate the surgery with acceptable risk. The second question rests on performing a GA for individual decision-making in order to establish the degree of patient frailty. In ovarian cancer, however, patients presenting with advanced disease may be debilitated owing to the effects of the disease, such as weight loss and hypoalbuminemia. Patients who need time to be medically optimized prior to surgery or who are initially unresectable on presentation may benefit from neoadjuvant therapy.

## Renal tumors

A common finding in older patients is small renal masses found incidentally on CT scans. The majority of small renal masses are malignant, but about 60% are indolent cancers, while 20% are aggressive cancers ^28^. In older patients, particularly if the patient is frail, the finding of a small mass in the kidney poses a challenge. A biopsy may be used to risk-stratify patients in order to avoid unnecessary surgical interventions. However, renal cell masses have a high chance of harboring a renal cell carcinoma. Alternative approaches to performing a biopsy are active surveillance, partial nephrectomy, radical nephrectomy, cryoablation, and radiofrequency ablation. In patients with frailty and comorbidities, the risk of dying of causes other than renal cell cancer is increased. Thus, a GA is recommended before proceeding with either a renal biopsy or other treatment options. Studies show that both partial nephrectomy and radical nephrectomy provide excellent oncologic results ^28^. Partial nephrectomy or nephron-sparing surgery reduces the incidence of renal dysfunction, but it is unclear whether these approaches improve survival. An alternative approach is active surveillance. There is no accepted protocol for active surveillance, but, according to the studies that have been performed, this approach is a non-inferior management strategy with regard to oncologic outcome for selected patients, depending on both disease characteristics and patient characteristics. Thermal ablative techniques such as cryoablation and radiofrequency ablation are alternatives to surgery. These methods are inferior to surgery for oncological outcome, with a meta-analysis showing that 5.2% of lesions managed with cryoablation and 12.9% of lesions managed with radiofrequency ablation had local progression after a median of 18.7 months ^29^. This approach may be considered in frail patients who are not candidates for surgery.

Cytoreductive nephrectomy is considered standard of care in patients with metastatic renal cell carcinoma ^30^. Surgery may be efficient in treating symptoms associated with the tumor, such as hematuria and pain. Studies regarding surgery for metastatic renal cell carcinoma in older patients have not included GA variables, and we have little knowledge of the impact of frailty on postoperative outcomes ^31^. Older patients should undergo surgery if they are considered fit and the tumor is resectable, and it is recommended that surgery is followed by systemic targeted therapies.

## Central nervous system tumors

Unfortunately, most studies in CNS tumors in older patients lack data regarding frailty. Poon and colleagues found that only 5 out of 13 studies in older patients with meningiomas reported data on comorbidity ^32^. In the review by Poon, it is emphasized that meningiomas are benign and that surgery can potentially achieve excellent long-term outcomes in older patients as long as the risks of surgery are acceptable. In small or medium-sized meningiomas, stereotactic radiosurgery is recommended. Data on the watchful waiting strategy in patients not treatable with stereotactic radiosurgery are very limited.

In high-grade gliomas (anaplastic astrocytoma, oligodendroglioma, and glioblastoma multiforme [GBM]), the prognosis is poor, and the optimal extent of surgical resection in older patients remains uncertain. A systematic review by Almenawer and colleagues concluded that outcomes such as overall survival and functional status were positively associated with increasing extents of safe resection, but the number of patients over the age of 75 or 80 in the included studies is not reported, nor are GA or frailty data ^33^. In a study by Arvold about treatment options for GBM in older patients, age is reported to be the most powerful prognostic factor, while the extent of surgical resection is the most important predictor of survival ^34^. In general, there are very little data on surgery for older patients with GBM. A review by Gallego and colleagues looked at the management of older adults with low- and high-grade gliomas ^35^. They also concluded that more prospective studies are needed, but studies indicate that active management of older patients with gliomas may have a positive impact on survival without impairing either cognition or QoL. There has been a shift from a nihilist therapeutic attitude towards this population to an active evidence-based approach. In patients with GBM, the need for histology in order to establish the correct classification and grading is emphasized, as magnetic resonance imaging is not sufficient. Although a modest improvement in survival with surgical resection compared to biopsy is seen, the benefit of resection decreases in the presence of poor performance status, neurological deficits, larger tumors, and patients with chronic obstructive pulmonary disease ^36^.

## Conclusions

Our knowledge regarding the optimal surgical treatment of older patients with cancer is slowly increasing, but most studies are still reporting data from a selected group of fit older patients. It is important to emphasize that fit older patients seem to tolerate surgery well, while patients who are frail experience more complications and poorer survival. However, we need more data regarding the treatment of patients with vulnerability, frailty, comorbidities, functional limitations, and cognitive impairment. Such data are essential in order to translate results from clinical studies into clinical practice, and we believe that it should be mandatory to include frailty data in all studies of older cancer patients.

## Abbreviations

ASA, American Society of Anesthesiology; CNS, central nervous system; CRC, colorectal cancer; GA **,** geriatric assessment; GBM, glioblastoma multiforme; QoL, quality of life.

## References

[1] Handforth C Clegg A Young C: The prevalence and outcomes of frailty in older cancer patients: a systematic review. *Ann Oncol.* 2015;26(6):1091–101. 10.1093/annonc/mdu540 25403592

[2] Ommundsen N Wyller TB Nesbakken A: Frailty is an independent predictor of survival in older patients with colorectal cancer. *Oncologist.* 2014;19(12):1268–75. 10.1634/theoncologist.2014-0237 25355846 PMC4257747

[3] PACE participants, Audisio RA Pope D: Shall we operate? Preoperative assessment in elderly cancer patients (PACE) can help. A SIOG surgical task force prospective study. *Crit Rev Oncol Hematol.* 2008;65(2):156–63. 10.1016/j.critrevonc.2007.11.001 18082416

[4] Walter LC Covinsky KE: Cancer screening in elderly patients: a framework for individualized decision making. *JAMA.* 2001;285(21):2750–6. 10.1001/jama.285.21.2750 11386931

[5] Kristjansson SR Nesbakken A Jordhøy MS: Comprehensive geriatric assessment can predict complications in elderly patients after elective surgery for colorectal cancer: a prospective observational cohort study. *Crit Rev Oncol Hematol.* 2010;76(3):208–17. 10.1016/j.critrevonc.2009.11.002 20005123

[6] Puts MT Santos B Hardt J: An update on a systematic review of the use of geriatric assessment for older adults in oncology. *Ann Oncol.* 2014;25(2):307–15. 10.1093/annonc/mdt386 24256847

[7] Feng MA McMillan DT Crowell K: Geriatric assessment in surgical oncology: a systematic review. *J Surg Res.* 2015;193(1):265–72. 10.1016/j.jss.2014.07.004 25091339 PMC4267910

[8] Fagard K Leonard S Deschodt M: The impact of frailty on postoperative outcomes in individuals aged 65 and over undergoing elective surgery for colorectal cancer: A systematic review. *J Geriatr Oncol.* 2016;7(6):479–91. 10.1016/j.jgo.2016.06.001 27338516

[9] Huisingh-Scheetz M Walston J: How should older adults with cancer be evaluated for frailty? *J Geriatr Oncol.* 2017;8(1):8–15. 10.1016/j.jgo.2016.06.003 27318797 PMC5161734

[10] Fried LP Tangen CM Walston J: Frailty in older adults: evidence for a phenotype. *J Gerontol A Biol Sci Med Sci.* 2001;56(3):M146–56. 10.1093/gerona/56.3.M146 11253156

[11] Mitnitski AB Mogilner AJ Rockwood K: Accumulation of deficits as a proxy measure of aging. *ScientificWorldJournal.* 2001;1:323–36. 10.1100/tsw.2001.58 12806071 PMC6084020

[12] Scher KS Hurria A: Under-representation of older adults in cancer registration trials: known problem, little progress. *J Clin Oncol.* 2012;30(17):2036–8. 10.1200/JCO.2012.41.6727 22547597

[13] Hamaker ME Schiphorst AH Verweij NM: Improved survival for older patients undergoing surgery for colorectal cancer between 2008 and 2011. *Int J Colorectal Dis.* 2014;29(10):1231–6. 10.1007/s00384-014-1959-y 25024043

[14] Hamaker ME Prins MC Schiphorst AH: Long-term changes in physical capacity after colorectal cancer treatment. *J Geriatr Oncol.* 2015;6(2):153–64. 10.1016/j.jgo.2014.10.001 25454769

[15] Papamichael D Audisio RA Glimelius B: Treatment of colorectal cancer in older patients: International Society of Geriatric Oncology (SIOG) consensus recommendations 2013. *Ann Oncol.* 2015;26(3):463–76. 10.1093/annonc/mdu253 25015334

[16] Ugolini G Ghignone F Zattoni D: Personalized surgical management of colorectal cancer in elderly population. *World J Gastroenterol.* 2014;20(14):3762–77. 10.3748/wjg.v20.i14.3762 24833841 PMC3983435

[17] Rønning B Wyller TB Nesbakken A: Quality of life in older and frail patients after surgery for colorectal cancer-A follow-up study. *J Geriatr Oncol.* 2016;7(3):195–200. 10.1016/j.jgo.2016.03.002 27067579

[18] Schiergens TS Lindenthaler A Thomas MN: Time-dependent impact of age and comorbidities on long-term overall survival after liver resection. *Liver Int.* 2016;36(9):1340–50. 10.1111/liv.13068 26778517

[19] Booth CM Nanji S Wei X: Management and Outcome of Colorectal Cancer Liver Metastases in Elderly Patients: A Population-Based Study. *JAMA Oncol.* 2015;1(8):1111–9. 10.1001/jamaoncol.2015.2943 26355283

[20] Beltrame V Gruppo M Pastorelli D: Outcome of pancreaticoduodenectomy in octogenarians: Single institution's experience and review of the literature. *J Visc Surg.* 2015;152(5):279–84. 10.1016/j.jviscsurg.2015.06.004 26117303

[21] Riall TS Sheffield KM Kuo YF: Resection benefits older adults with locoregional pancreatic cancer despite greater short-term morbidity and mortality. *J Am Geriatr Soc.* 2011;59(4):647–54. 10.1111/j.1532-5415.2011.03353.x 21453378 PMC3156667

[22] Casadei R Ricci C Lazzarini E: Pancreatic resection in patients 80 years or older: a meta-analysis and systematic review. *Pancreas.* 2014;43(8):1208–18. 10.1097/MPA.0000000000000182 25333405

[23] Elit L: Cervical cancer in the older woman. *Maturitas.* 2014;78(3):160–7. 10.1016/j.maturitas.2014.04.018 24861965

[24] Tew WP Fleming GF: Treatment of ovarian cancer in the older woman. *Gynecol Oncol.* 2015;136(1):136–42. 10.1016/j.ygyno.2014.10.028 25448455

[25] Di Donato V Kontopantelis E Aletti G: Trends in Mortality After Primary Cytoreductive Surgery for Ovarian Cancer: A Systematic Review and Metaregression of Randomized Clinical Trials and Observational Studies. *Ann Surg Oncol.* 2017;24(6):1688–97. 10.1245/s10434-016-5680-7 27896508

[26] Moore KN Reid MS Fong DN: Ovarian cancer in the octogenarian: does the paradigm of aggressive cytoreductive surgery and chemotherapy still apply? *Gynecol Oncol.* 2008;110(2):133–9. 10.1016/j.ygyno.2008.03.008 18495221

[27] Lichtman SM: How I treat ovarian cancer in older women. *J Geriatr Oncol.* 2014;5(3):223–9. 10.1016/j.jgo.2014.06.001 24954887

[28] Mirza M: Management of Small Renal Masses in the Older Adult. *Clin Geriatr Med.* 2015;31(4):603–13. 10.1016/j.cger.2015.06.005 26476119

[29] Kunkle DA Uzzo RG: Cryoablation or radiofrequency ablation of the small renal mass : a meta-analysis. *Cancer.* 2008;113(10):2671–80. 10.1002/cncr.23896 18816624 PMC2704569

[30] Zustovich F Novara G: Advanced kidney cancer: treating the elderly. *Expert Rev Anticancer Ther.* 2013;13(12):1389–98. 10.1586/14737140.2013.846095 24224483

[31] Sun M Abdollah F Schmitges J: Cytoreductive nephrectomy in the elderly: a population-based cohort from the USA. *BJU Int.* 2012;109(12):1807–12. 10.1111/j.1464-410X.2011.10569.x 21951647

[32] Poon MT Fung LH Pu JK: Outcome of elderly patients undergoing intracranial meningioma resection--a systematic review and meta-analysis. *Br J Neurosurg.* 2014;28(3):303–9. 10.3109/02688697.2013.841857 24073759

[33] Almenawer SA Badhiwala JH Alhazzani W: Biopsy versus partial versus gross total resection in older patients with high-grade glioma: a systematic review and meta-analysis. *Neuro Oncol.* 2015;17(6):868–81. 10.1093/neuonc/nou349 25556920 PMC4483123

[34] Arvold ND Reardon DA: Treatment options and outcomes for glioblastoma in the elderly patient. *Clin Interv Aging.* 2014;9:357–67. 10.2147/CIA.S44259 24591820 PMC3937103

[35] Gállego Pérez-Larraya J Delattre JY: Management of elderly patients with gliomas. *Oncologist.* 2014;19(12):1258–67. 10.1634/theoncologist.2014-0170 25342314 PMC4257742

[36] Laperriere N Weller M Stupp R: Optimal management of elderly patients with glioblastoma. *Cancer Treat Rev.* 2013;39(4):350–7. 10.1016/j.ctrv.2012.05.008 22722053

